# Gender differences in spinal mobility during postural changes: a detailed analysis using upright CT

**DOI:** 10.1038/s41598-024-59840-8

**Published:** 2024-04-21

**Authors:** Ryo Mizukoshi, Mitsuru Yagi, Yoshitake Yamada, Yoichi Yokoyama, Minoru Yamada, Kota Watanabe, Masaya Nakamura, Takeo Nagura, Masahiro Jinzaki

**Affiliations:** 1 Department of Orthopaedic Surgery, Keio University School of Medicine, 35 Shinanomachi Shinjyuku, Tokyo, Japan; 2 Department of Orthopaedic Surgery, School of Medicine, International University of Health and Welfare, Chiba, Japan; 3 Department of Radiology, Keio University School of Medicine, 35 Shinanomachi Shinjyuku, Tokyo, Japan

**Keywords:** Gender differences, Spinal mobility, Postural changes, Upright CT, Lumbar alignment, Anatomy, Medical research

## Abstract

Lumbar spinal alignment is crucial for spine biomechanics and is linked to various spinal pathologies. However, limited research has explored gender-specific differences using CT scans. The objective was to evaluate and compare lumbar spinal alignment between standing and sitting CT in healthy individuals, focusing on gender differences. 24 young and 25 elderly males (M) and females (F) underwent standing and sitting CT scans to assess lumbar spinal alignment. Parameters measured and compared between genders included lumbar lordosis (LL), sacral slope (SS), pelvic tilt (PT), pelvic incidence (PI), lordotic angle (LA), foraminal height (FH), and bony boundary area (BBA). Females showed significantly larger changes in SS and PT when transitioning from standing to sitting (*p* = .044, *p* = .038). A notable gender difference was also observed in the L4-S LA among the elderly, with females showing a significantly larger decrease in lordotic angle compared to males (− 14.1° vs. − 9.2°, *p* = .039*). Females consistently exhibited larger FH and BBA values, particularly in lower lumbar segments, which was more prominent in the elderly group (M vs. F: L4/5 BBA 80.1 mm^2^ [46.3, 97.8] vs. 109.7 mm^2^ [74.4, 121.3], *p* = .019 in sitting). These findings underline distinct gender-related variations in lumbar alignment and flexibility, with a focus on noteworthy changes in BBA and FH in females. Gender differences in lumbar spinal alignment were evident, with females displaying greater pelvic and sacral mobility. Considering gender-specific characteristics is crucial for assessing spinal alignment and understanding spinal pathologies. These findings contribute to our understanding of lumbar spinal alignment and have implications for gender-specific spinal conditions and treatments.

## Introduction

In recent years, the dynamics of spinal alignment have received significant attention in medical research. One aspect that has proven crucial in these studies is the analysis of spinal changes under various loads. Traditionally, image analyses under load have been conducted through standing MRI and loaded CT. However, several factors, such as narrow FOV, long imaging time, and low resolution, have made the use of standing MRI problematic for morphological evaluation^1^. On the other hand, loaded CT, while able to replicate pseudo standing load in a supine position, lacks physiological accuracy due to the differences from natural standing in terms of gravity direction and muscle activity^2^.

While X-rays or EOS have been extensively used in reporting and evaluations made in positions like standing and sitting, the field lacks evaluations using loaded CT^3–9^. Furthermore, while differences in alignment and postural changes according to gender and age have been previously reported, there has been a glaring absence of detailed evaluation using CT^4,7,8^.

Through CT evaluation, researchers can assess the intervertebral foramina and the facet joints unlike with X-rays. There is also evidence to suggest that lumbar degenerative spondylolisthesis is more common in female, particularly at L4/5 levels, indicating the existence of diseases where the pathology varies between men and female^10^.

Recognizing these gaps, this study aims to leverage upright CT to evaluate spinal alignment and lumbar components under physiological load during standing and sitting among healthy volunteers. Our goal was to delineate the postural changes between men and female in detail, shedding new light on spinal mobility and its implications in medical diagnostics and treatment plans.

## Materials and methods

### Study design

This is a cross-sectional study. Images were captured in the standing and relaxed sitting positions using upright CT. The subjects comprised 24 young healthy volunteers (aged 21–40, mean age 30.3 ± 5.7 years, 13 males, 11 females) and 25 elderly individuals (aged 61–79, mean age 65.7 ± 3.8 years, 12 males, 13 females). The criteria for being considered healthy included not having back pain, not smoking, having a body mass index (BMI) between 18.5 and 25 kg/m^2^, no history of spinal surgery, and not being pregnant or potentially pregnant.

### Image acquisition

All subjects were imaged in the standing and relaxed sitting positions using upright CT. For the standing posture, images were taken without leaning on the back rod installed in the upright CT, standing as much as to avoid swaying. For the relaxed sitting position, a plate was set up within the upright CT to allow subjects to sit, with hip and knee joints at 90 degrees, and without using a backrest. Uniformity of posture was managed by an orthopedic doctor and a radiologist present during the imaging process. Before the imaging session, the orthopedic doctor and radiologist demonstrated the required postures to the participants, ensuring they understood exactly how to position their bodies. Both professionals were present to visually inspect and adjust the participants' postures before each imaging session commenced. This direct supervision ensured that the subjects' hip and knee joints were at the correct 90° angle for sitting positions and that their standing posture was as required for the study. The orthopedic doctor and radiologist provided clear, verbal instructions to participants on how to assume the required postures. They also assisted participants in achieving these postures when necessary, making minor adjustments to ensure accuracy. Throughout the imaging process, these specialists performed consistency checks across all participants to ensure that the posture maintained met the study's strict guidelines. Upright CT (prototype TSX-401R, Canon Medical Systems) images were acquired under the same conditions: tube current, 10–350 mA (using noise index 24 for slice thickness 5 mm); rotation speed, 0.5 s; and slice thickness, 0.5 mm. Images were reconstructed using Adaptive Iterative Dose Reduction 3D (Canon Medical Systems, Ohtawara, Japan) to reduce radiation dose^11,12^.

The scan range included the outer ear canal to the femoral head, including the femur. The total average effective dose of upright CT (standing + sitting CT) in this study was about 8 mSv.

The present study was approved by the Ethics Committee of Keio University School of Medicine. It was conducted in accordance with the Helsinki Declaration and all prescribed protocols. Moreover, an informed consent was obtained from all participants.

### Radiographic measurements

All data were analyzed using commercial software (Zed View 14.0.0; LEXI Co., Ltd.). CT images were reconstructed in MPR, and spine-pelvis parameters were measured using the projected data. Lumbar lordosis (LL), sacral slope (SS), pelvic tilt (PT), pelvic incidence (PI), and intervertebral angles were measured^13^. IVA was defined as the angle in the sagittal plane between the lower endplate of the upper vertebral body and the upper endplate of the lower vertebral body, with forward bending being positive and backward bending negative, in relation to the axis passing through the center of the femoral head. The lordotic angle of the lumbar spine, also referred to as the L1-S to L5-S segment angle, is defined as the angle formed between the superior endplate of the first sacral vertebra (S1) and the inferior endplate of the first to fifth lumbar vertebra (L1-5). In particular, L4-S is a measure of lordosis, present in the lower section of the lumbar spine at the junction where the spine meets the sacrum. It is an important parameter for assessing the biomechanical alignment of the spine and can have implications for posture.

Morphological evaluation of the intervertebral foramen was performed by measuring the foraminal height (FH), and bony boundary area (BBA). These measurements were taken on both sides of the intervertebral foramen in the standing and sitting positions and the average or the average difference for each side was calculated.

#### FH

**Figure 1:**
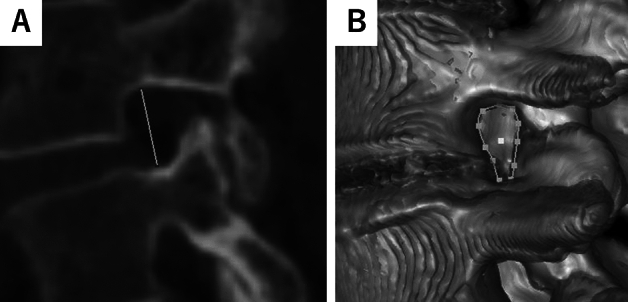
Representative image of foraminal height and bone boundary area. (**A**) Representative Axial View of L4/L5 Foramen. The solid line shows the foraminal height (FH). FH is defined as the maximum distance between the lower edge of the superior pedicle and the upper edge of the inferior pedicle. (**B**) 3D Reconstruction of L4/L5 Foramen. Dotted circle indicates the bone boundary area (BBA) of the foramen. BBA is defined as the area enclosed by the posterior edge of the vertebral body, the lower edge of the superior pedicle, the anterior edge of the inferior articular process, the anterior edge of the superior articular process, and the upper edge of the inferior pedicle.

FH is defined as the maximum distance between the lower edge of the superior pedicle and the upper edge of the inferior pedicle in the sagittal plane, we pointed out the bottom center and top center of the pedicle and measured the distance (Fig. 1A)^14^.

#### BBA

BBA is defined as the area enclosed by the posterior edge of the vertebral body, the lower edge of the superior pedicle, the anterior edge of the inferior articular process, the anterior edge of the superior articular process, and the upper edge of the inferior pedicle. Due to rotation of the vertebral body altering the positions of the anterior and posterior edges when defined in the sagittal plane, we instead assessed the area on the plane formed by the axis of the FH and the axis of the inferior pedicle. We referred to a 3D model created by Zed View and adjusted the area enclosed by the bone to define the BBA (Fig. 1B)^15^.

### Intraclass correlation coefficient values for various spinal parameters and measurements

The intraclass correlation coefficient (ICC) of the intra- and inter-observer reliabilities of measurements obtained from CT images of 10 randomly selected patients were calculated. We classified the ICC values according to the criteria introduced by Aubin et al.; < 0.24, 0.25–0.49, 0.50–0.69, 0.70–0.89, and 0.90–1.0 were considered to be poor, low, fair to moderate, good, good to excellent, respectively. Two board certified orthopedic surgeons independently measured all radiograph parameters. One examiner measured once and the other examiner measured twice a week apart after brief lecture for standardize the measurement. Examiners were blinded to patient clinical information and other measurement.

### Statistical analysis

All statistical analyses were performed using R Version 4.2.1. Radiographic measurement data were expressed as median (1st–3rd quartile in parentheses). The level of significance was set at *p* < 0.05. Differences in posture within the same individual were tested using the Wilcoxon signed-rank test, and differences in posture between ages and sexes were tested using the Mann–Whitney U test.

## Results

### Reliability assessment of spinal measurements via CT

The intra-observer ICC values for spinal measurements ranged from 0.855 to 0.996, showcasing excellent consistency. Similarly, the inter-observer ICC values exhibited excellent reliability, falling within a range of 0.87–0.98. The parameters FH and BBA also demonstrated good reliability, with intra-observer ICC values of 0.855 and 0.982, and inter-observer ICC values of 0.805 and 0.938, respectively (Supplemental Table 1). These findings highlight the high reproducibility of spinal measurements conducted using CT.

### Summary of the patient cohort

Table 1 shows the background of the subjects. The average LL was 42.2°, SS averaged at 31.1°, PT averaged at 14.4°, and the average PI was 43.6°.

**Table 1:** Background of the study subjects.

Variables	Total	Young	Elderly	Males	Females
Gender (Male : Female)	25: 24	13: 11	12: 13	25: 0	0: 24
Age (year-old)	48.4 ± 18.3	30.3 ± 5.7	65.7 ± 3.8	47.4 ± 18.4	49.4 ± 18.2
Height (cm)	164.7 ± 8.5	167.0 ± 7.2	162.6 ± 9.1	170.5 ± 3.9	158.7 ± 7.7
Weight (kg)	59.5 ± 8.2	60.3 ± 7.6	58.7 ± 8.7	65.8 ± 4.7	53.0 ± 5.6
BMI (kg/m^2^)	21.8 ± 1.7	21.6 ± 1.8	22.1 ± 1.7	22.6 ± 1.6	21.0 ± 1.5
Sagittal spinal alignment					
Lumbar lordosis (LL, °)	42.2 (32.9, 48.7)	42.2 (33.9, 47.1)	43.4 (32.9, 52.4)	40.4 (29.0, 47.1)	46.6 (37.3, 54.3)
Sacral slope (SS, °)	31.1 (26.1, 37.5)	31.5 (27.2, 35.8)	31.1 (24.8, 39.0)	29.5 (23.2, 35.0)	33.5 (28.5, 38.3)
Pelvic tilt (PT, °)	14.4 (10.7, 17.7)	11.2 (8.3, 15.7)	15.7 (13.8, 20.9)	14.4 (9.5, 17.7)	14.4 (11.2, 17.7)
Pelvic incidence (PI, °)	43.6 (40.8, 55.3)	42.1 (35.9, 47.5)	48.5 (42.3, 56.1)	42.3 (36.5, 48.9)	47.7 (41.1, 55.5)

Means and standard deviations. Median, 1st–3rd quartile in parentheses.

### Comparisons of the change of regional lumbar spinal parameters by posture between male and female subjects

**Figure 2:**
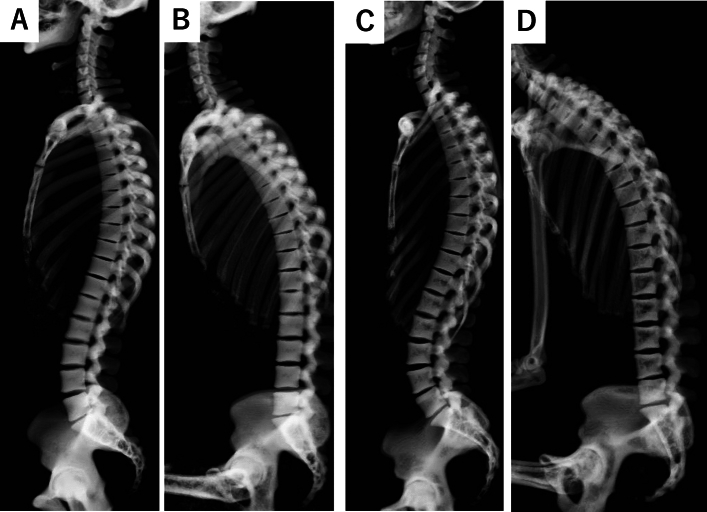
Representative cases of the change of spinal alignment by posture in the young subjects. (**A**) The standing whole spine CT of 32-year-old male. (**B**) The sitting whole spine CT of 32-year-old male. (**C**) The standing whole spine CT of 38-year-old female. (**D**) The sitting whole spine CT of 38-year-old female. A significant spinal alignment change was observed in a female patient when transitioning to a sitting position.

**Figure 3:**
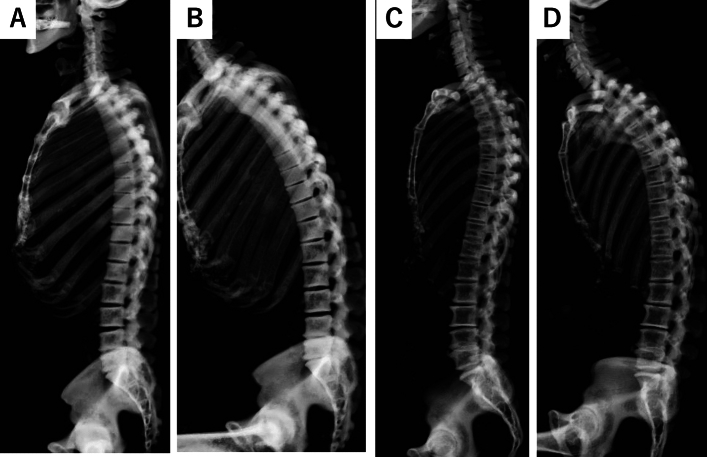
representative cases of the change of spinal alignment by posture in the elderly subjects. (**A**) The standing whole spine CT of 74-year-old male. (**B**) The sitting whole spine CT of 74-year-old male. (**C**) The standing whole spine CT of 72-year-old female. (**D**) The sitting whole spine CT of 72-year-old female. A significant spinal alignment change was observed in a female patient when transitioning to a sitting position.

The representative CT images were shown in Figs. 2 and 3. Females had a significantly larger LL than males during standing (46.6° vs. 40.4°, *p* = 0.03, Table 2). However, when transitioning to sitting, females exhibited a larger reduction rate in LL compared to males, though not statistically significant (− 91.4% vs. − 80.2%, *p* = 0.052, Table 2). Additionally, while there was a significant decrease in the reduction rate for elderly males, elderly females retained a similar reduction rate in LL compared to young females (− 90.9% vs. − 53.0% for males and − 95.8% vs. − 84.0% for females, *p* = 0.01, 0.29). (Supplemental Table 2).

**Table 2:** Comparisons of the change of regional lumbar spinal alignments by posture between male and female subjects.

Variables		Standing		Sitting		delta value		Reduction rate		*P*-value			
		Male	Female	Male	Female	Male	Female	Male (%)	Female (%)	*p*1	*p*2	*p*3	*p*4
LL (°)	Young	38.2 (32.2, 45.2)	46.8 (38.9, 50.5)	4.3 (− 8.6, 7..7)	2.7 (− 7.4, 19.3)	− 42.9 (− 46.0, − 33.8)	− 48.6 (− 52.5, − 34.0)	− 90.9 (− 82.9, − 120.4)	− 95.8 (− 59.3, − 116.6)	0.04*	0.25	0.1	0.55
	Elderly	42.8 (28.9, 50.5)	44.4 (37.9, 60.1)	19.4 (10.5, 23.0)	7.1 (− 4.6, 32.8)	− 18.7 (− 29.5, − 11.9)	− 37.3 (− 42.5, − 20.9)	− 53.0 (− 29.0, − 75.6)	− 84.0 (− 53.9, − 112.1)	0.17	0.16	0.1	0.01*
	Whole	40.4 (29.0, 47.1)	46.6 (37.3, 54.3)	7.7 (0.5, 18.3)	4.4 (− 6.5, 20.9)	− 31.2 (− 42.9, − 17.0)	− 40.5 (− 49.5, − 26.6)	− 80.2 (− 48.1, 98.3)	− 91.4 (58.0, 115.4)	0.03*	0.61	0.06	0.05
SS (°)	Young	29.0 (26.1, 32.7)	35.7 (30.6, 36.8)	5.6 (4.2, 14.6)	9.6 (3.6, 19.4)	− 23.9 (− 27.8, − 15.7)	− 30.4 (− 34.6, − 18.1)	− 78.9 (− 56.4, 86.0)	− 79.3 (− 52.4, − 89.7)	0.06	0.41	0.11	0.41
	Elderly	33.0 (23.2, 37.4)	30.8 (28.3, 39.0)	22.3 (16.7, 25.4)	8.5 (0.1, 23.7)	− 10.1 (− 13.7, 2.7)	− 24.8 (− 28.8, − 10.9)	− 28.8 (− 1.7, − 50.2)	− 74.3 (− 51.5, − 96.4)	0.29	0.08	0.04*	0.06
	Whole	29.5 (23.2, 35.0)	33.5 (28.5, 38.3)	14.6 (5.6, 21.6)	9.1 (2.4, 20.8)	− 15.2 (− 25.5, − 7.7)	− 26.4 (− 30.9, − 15.5)	− 56.4 (23.7, 81.0)	− 76.1 (50.2, 92.4)	0.07	0.15	0.04*	0.17
PT (°)	Young	10.7 (5.5, 15.6)	11.8 (11.1, 15.1)	34.7 (28.9, 37.0)	44.1 (35.2, 51.4)	24.4 (17.2, 31.0)	33.0 (20.7, 37.7)	231.9 (136.5, 312.7)	267.6 (208.8, 326.2)	0.19	0.08	0.09	0.63
	Elderly	15.3 (14.0, 20.9)	16.6 (13.6, 20.9)	29.0 (21.8, 33.2)	42.3 (28.2, 51.3)	12.7 (0.6, 15.7)	25.7 (10.8, 30.4)	82.1 (10.9, 126.0)	133.1 (56.9, 174.1)	0.39	0.02*	0.04*	0.06
	Whole	14.4 (9.5, 17.7)	14.4 (11.2, 17.7)	30.6 (23.9, 36.8)	43.2 (33.1, 51.9)	16.2 (9.8, 28.39	28.7 (15.5, 33.1)	136.5 (69.8, 231.9)	178.4 (97.7, 262.6)	0.23	0.01*	0.04*	0.2
PI (°)	Young	39.8 (33.4, 43.4)	43.1 (41.1, 52.4)	40.3 (33.7, 45.3)	48.8 (44.2, 55.9)	2.5 (0.8, 3.1)	2.4 (1.7, 4.5)	6.4 (2.2, 8.1)	6.0 (4.4, 9.3)	0.06	0.04*	0.49	0.35
	Elderly	41.5 (34.8, 43.6)	49.7 (43.3, 56.1)	49.5 (42.9, 58.5)	51.3 (46.7, 57.1)	1.3 (0.6, 3.2)	1.1 (0.7, 2.8)	2.7 (1.2, 7.3)	2.2 (1.5, 4.6)	0.27	0.25	0.78	0.31
	Whole	42.3 (36.5, 48.9)	47.7 (41.1, 55.5)	45.1 (37.3, 50.4)	50.5 (44.7, 57.0)	2.3 (0.7, 3.1)	1.9 (0.8, 3.5)	4.1 (1.3, 8.0)	4.3 (1.7, 7.8)	0.05*	0.04*	0.79	0.31

Median. 1st–3rd quartile in parentheses. *Indicates statistically significant. *P*1 indicates the comparisons of the values between males and females during standing. *P*2 indicates the comparisons of the values between males and females during sitting. *P*3 indicates the comparisons between the delta value of males and females. *P*4 indicates the comparisons between the reduction rate of males and females.

For SS, the study found significantly larger delta values in females compared to males among both the overall participant group and the elderly subset. Specifically, the difference was − 23.9° in young males versus − 30.4° in females, and − 10.1° in elderly males versus − 24.8° in elderly females, and − 15.2° in males versus − 26.4° in females for the overall group, with *P*-values of 0.11, 0.04 and 0.04, respectively. Similarly, for PT, significantly larger delta values were observed in females compared to males in both the overall participant group and the elderly subset. The delta values were 24.4° in young males versus 33.0° in young females, and 12.7° in elderly males versus 25.7° in elderly females 16.2° in males versus 28.7° in females for the overall group, with *P*-values of 0.09, 0.04 and 0.04, respectively. Lastly, PI was significantly larger in females than in males, with values of 42.3° for males and 47.7° for females, and a *P*-value of 0.048 (Table 2). Overall, these findings suggest that females exhibited a larger lordosis to match their higher PI and retained this flexibility until their elderly years.

### Comparisons of the change of intervertebral disc angle and lordotic angles by posture between male and female subjects

There were no statistically significant changes in intervertebral disc angle between standing and sitting postures for both genders and across different age groups at all lumbar disc levels (Table 3). However, for the L4/5 disc level, there was a trend towards a larger delta in females, though not statistically significant (− 8.2° vs. − 10.4°, *p* = 0.28, Table 3). Furthermore, when comparing the lordotic angle of the lower lumbar spine (L3-S, L4-S segment), a significantly large delta was observed in elderly females when compared with elderly males (− 11.3° vs. − 21.5°, *p* = 0.04) (− 9.3° vs. − 14.1°, *p* = 0.04) (Table 4).

**Table 3:** Comparisons of the change of intervertebral disc angle by posture between male and female subjects.

Variables		Standing		Sitting		Delta value		*P*-value		
		Male	Female	Male	Female	Male	Female	*p*1	*p*2	*p*3
L1/2 (°)	Young	5.4 (4.2, 6.4)	4.6 (2.5, 6.4)	1.1 (− 1.7, 2.0)	0.2 (− 1.7, 5.3)	− 4.7 (− 6.6, − 2.2)	− 4.2 (− 6.4, 0.1)	0.27	0.44	0.36
	Elderly	5.6 (5.1, 7.9)	6.5 (5.6, 7.2)	2.1 (0.9, 6.4)	3.3 (2.5, 3.7)	− 2.0 (− 4.1, − 0.1)	− 3.3 (− 4.8, − 1.9)	0.43	0.79	0.43
	Whole	5.5 (4.6, 6.6)	6.2 (4.3, 6.9)	1.3 (0.4, 2.4)	2.6 (0, 5.0)	− 3.3 (− 5.9, − 1.7)	− 3.7 (− 5.3, − 0.2)	0.76	0.22	0.8
L2/3 (°)	Young	7.1 (6.4, 8.1)	8.1 (7.1, 9.9)	0 (− 0.9, 1.1)	− 3.1 (− 3.3, 5.8)	− 7.8 (− 9.3, − 4.3)	− 5.0 (− 11.4, − 2.9)	0.1	0.48	0.78
	Elderly	6.7 (5.6, 9.4)	7.7 (4.5, 9.4)	3.5 (1.5, 7.1)	1.1 (− 0.2, 7.4)	− 2.8 (− 4.4, − 0.4)	− 3.2 (− 6.1, − 0.9)	0.85	0.22	0.62
	Whole	7.1 (5.9, 9.1)	8.0 (6.2, 9.4)	1.2 (− 0.2, 3.5)	0.7 (− 3.1, 6.3)	− 4.3 (− 7.9, − 2.5)	− 4.8 (− 9.9, − 1.4)	0.17	0.61	0.95
L3/4 (°)	Young	7.7 (6.1, 9.6)	8.1 (7.1, 9.9)	− 0.6 (− 1.9, 0)	− 0.9 (− 2.5, 3.8)	− 9.6 (− 10.1, − 7.3)	− 10.3 (− 11.2, − 6.5)	0.02	0.45	0.54
	Elderly	8.6 (6.8, 10.8)	7.8 (6.9, 9.5)	3.4 (1.5, 6.2)	2.2 (− 2.4, 3.2)	− 4.3 (− 8.0, − 11.6)	− 5.6 (− 9.4, − 2.0)	0.26	0.11	0.45
	Whole	7.7 (6.1, 9.7)	8.9 (7.3, 10.6)	0.4 (− 0.6, 2.6)	0 (− 2.4, 3.6)	− 7.4 (− 9.6, − 4.2)	− 7.7 (− 10.8, − 3.4)	0.22	0.62	0.54
L4/5 (°)	Young	9.4 (7.3, 10.5)	10.5 (10.0, 11.2)	− 1.1 (− 2.0, 0.2)	− 1.9 (− 4.0, − 0.9)	− 10.4 (− 12.3, − 8.6)	− 12.5 (− 14.0, − 10.7)	0.08	0.12	0.19
	Elderly	7.9 (2.5, 10.2)	7.7 (4.9, 11.2)	3.1 (2.8, 5.0)	2.0 (− 4.6, 5.8)	− 3.3 (− 7.5, − 0.8)	− 4.9 (− 9.3, − 2.5)	0.89	0.12	0.41
	Whole	9.4 (5.4, 10.5)	10.0 (7.3, 11.2)	1.5 (− 1.1, 3.1)	− 1.3 (− 4.4, 2.6)	− 8.2 (− 11.3, − 3.3)	− 10.4 (− 13.0, − 4.6)	0.2	0.07	0.28
L5/S1 (°)	Young	9.1 (5.7, 11.1)	9.0 (6.0, 10.5)	2.4 (1.6, 4.3)	2.0 (1.4, 3.5)	− 6.1 (− 8.9, − 4.5)	− 4.8 (− 9.2, − 3.4)	0.86	0.75	0.71
	Elderly	14.8 (10.4, 16.1)	13.0 (7.0, 15.7)	9.6 (6.6, 12.0)	4.4 (3.4, 7.7)	− 5.0 (− 6.3, − 2.1)	− 6.2 (− 7.8, − 3.9)	0.25	0.06	0.25
	Whole	10.8 (6.8, 14.7)	9.2 (6.3, 13.3)	4.3 (2.2, 9.6)	3.5 (1.4, 5.6)	− 5.4 (− 7.9, − 3.5)	− 6.1 (− 8.4, − 3.8)	0.7	0.18	0.75

Median. 1st–3rd quartile in parentheses. *Indicates statistically significant. P1 indicates the comparisons of the values between males and females during standing.

P2 indicates the comparisons of the values between males and females during sitting.

P3 indicates the comparisons between the delta value of males and females.

**Table 4:** Comparisons of the Change of Lordic Angle by Posture between Male and Female Subjects.

Variables		Standing		Sitting		Delta value		*P*-value		
		Male	Female	Male	Female	Male	Female	*p*1	*p*2	*p*3
L1-S (°)	Young	43.6 (36.6, 48.1)	51.5 (40.6, 58.1)	4.8 (− 4.9, 8.1)	6.1 (− 4.3, 25.6)	− 42.2 (− 45.8, − 35.5)	− 48.4 (− 52.1, − 35.1)	0.02*	0.16	0.21
	Elderly	47.8 (36.1, 57.9)	48.1 (41.1, 58.4)	26.2 (10.6, 30.8)	10.5 (2.2, 36.5)	− 18.9 (− 31.1, − 10.8)	− 32.9 (− 40.9, − 16.9)	0.31	0.14	0.14
	Whole	47.4 (36.6, 49.2)	50.4 (40.8, 59.1)	10.6 (3.5, 24.6)	8.3 (0.7, 28.6)	− 34.3 (− 44.3, − 18.2)	− 37.8 (− 49.1, − 25.9)	0.04*	0.88	0.2
L2-S (°)	Young	41.1 (35.0, 44.8)	47.2 (42.4, 54.6)	4.6 (− 1.3, 8.6)	5.0 (1.9, 20.9)	− 37.9 (− 41.7, − 32.5)	− 39.6 (− 45.9, − 36.0)	0.02*	0.15	0.23
	Elderly	43.6 (31.9, 50.4)	44.8 (35.2, 54.7)	25.4 (15.5, 32.0)	9.0 (4.6, 36.5)	− 13.8 (− 26.6, − 9.4)	− 27.2 (− 33.7, − 12.6)	0.28	0.1	0.12
	Whole	42.7 (33.6, 45.7)	46.8 (38.9, 54.7)	13.7 (4.6, 25.7)	8.0 (3.8, 24.3)	− 28.8 (− 41.1, − 12.9)	− 33.2 (− 42.1, − 24.3)	0.04*	0.81	0.16
L3-S (°)	Young	34.1 (31.1, 36.6)	38.9 (37.2, 43.0)	4.7 (2.1, 11.4)	5.0 (2.8, 13.6)	− 29.1 (− 34.3, − 24.3)	− 32.7 (− 36.0, − 30.2)	0.01*	0.33	0.09
	Elderly	38.5 (27.0, 43.3)	38.5 (27.7, 42.6)	22.4 (16.1, 30.4)	12.5 (6.9, 28.5)	− 11.3 (− 19.0, − 7.4)	− 21.5 (− 28.3, − 12.3)	0.37	0.06	0.04*
	Whole	35.9 (27.3, 40.0)	38.7 (34.3, 43.0)	12.0 (4.7, 24.8)	8.8 (4.8, 17.2)	− 20.5 (− 32.2, − 10.7)	− 28.2 (− 33.0, − 20.2)	0.06	0.27	0.07
L4-S (°)	Young	23.2 (20.6, 28.0)	24.0 (21.9, 26.8)	6.7 (2.3, 8.5)	3.0 (1.1, 8.5)	− 19.6 (− 23.1, − 12.9)	− 20.5 (− 24.7, − 17.0)	0.35	0.22	0.271
	Elderly	32.9 (18.7, 34.3)	27.2 (22.9, 30.8)	21.2 (12.8, 24.5)	13.7 (8.3, 18.2)	− 9.3 (− 13.2, − 5.1)	− 14.1 (− 17.1, − 10.5)	0.26	0.049*	0.04*
	Whole	25.7 (19.5, 33.2)	25.3 (22.6, 29.7)	8.5 (5.7 21.9)	8.6 (2.9, 14.0)	− 12.9 (− 21.4, − 5.3)	− 17.0 (− 20.7, − 13.3)	0.45	0.12	0.1
L5-S (°)	Young	9.1 (5.7, 11.1)	9.0 (6.0, 10.5)	2.4 (1.6, 4.3)	2.0 (1.4, 3.5)	− 6.1 (− 8.9, − 4.5)	− 4.8 (− 9.2, − 3.4)	0.43	0.38	0.35
	Elderly	14.8 (10.4, 16.1)	13.0 (7.0, 15.7)	9.6 (6.6, 12.0)	4.4 (3.4, 7.7)	− 5.0 (− 6.3, − 2.1)	− 6.2 (− 7.8, − 3.9)	0.25	0.06	0.13
	Whole	10.8 (6.8, 14.7)	9.2 (6.3, 13.3)	4.3 (2.2, 9.6)	3.5 (1.4, 5.6)	− 5.4 (− 7.9, − 3.5)	− 6.1 (− 8.4, − 3.8)	0.35	0.18	0.37

Median. 1st–3rd quartile in parentheses. *Indicates statistically significant. *P*1 indicates the comparisons of the values between males and females during standing. *P*2 indicates the comparisons of the values between males and females during sitting. *P*3 indicates the comparisons between the delta value of males and females.

In summary, while there were no significant changes in intervertebral disc angles between standing and sitting postures, a trend towards larger changes in the L4/5 disc level of the females was observed. Additionally, there was a significant gender difference in the lordotic angle of the lower lumbar spine among elderly participants.

### Comparisons of the change of FH by posture between male and female subjects

At the upper disc levels, there were no significant differences in FH between males and females during standing and sitting (Table 5). At the L4/5 disc level, large delta for FH was observed in females when compared with males being statistically significant in elderly females and a trend observed in overall females (*p* = 0.28, 0.04).

**Table 5:** Comparisons of the change of foraminal height by posture between male and female subjects.

Variables		Standing		Sitting		Delta value		*P*-value		
		Male	Female	Male	Female	Male	Female	*p*1	*p*2	*p*3
L1/2 (mm)	Young	17.0 (16.8, 17.6)	16.9 (16.0, 17.5)	18.1 (17.8, 19.1)	17.4 (16.5, 18.3)	1.2 (0.8, 1.5)	0.7 (0.1, 1.4)	0.27	0.08	0.15
	Elderly	15.8 (14.5, 17.2)	15.9 (14.5, 16.8)	17.1 (15.0, 18.8)	17.0 (16.4, 17.7)	0.2 (0, 1.3)	1.6 (0.9, 1.9)	0.5	1	0.12
	Whole	16.8 (15.7, 17.6)	16.2 (15.3, 17.4)	17.9 (16.9, 19.1)	17.1 (16.4, 18.1)	1.0 (0.2, 1.5)	1.3 (0.1, 1.7)	0.52		
L2/3 (mm)	Young	16.9 (16.8, 18.0)	16.9 (16.3, 18.4)	19.8 (18.9, 20.2)	19.3 (18.4, 20.0)	2.0 (1.8, 2.7)	2.3 (1.3, 2.7)	0.33	0.16	0.82
	Elderly	16.5 (15.4, 18.5)	16.5 (15.7, 17.0)	18.3 (15.8, 20.0)	18.1 (16.9, 19.0)	0.7 (− 0.4, 1.8)	1.3 (0.7, 2.2)	0.34	0.35	0.21
	Whole	16.9 (16.1, 18.4)	16.5 (16.1, 17.5)	19.6 (18.1, 20.2)	18.6 (17.9, 19.5)	1.8 (0.8, 2.3)	1.8 (0.8, 2.5)	0.42	0.16	0.6
L3/4 (mm)	Young	17.5 (16.4, 17.9)	16.4 (15.7, 17.4)	19.9 (18.5, 20.2)	19.2 (19.0, 19.8)	2.8 (2.1, 3.2)	3.1 (1.9, 3.6)	0.16	0.26	0.69
	Elderly	16.3 (14.8, 17.9)	16.0 (15.9, 17.1)	17.8 (15.4, 20.4)	19.1 (17.0, 19.4)	0.7 (0.2, 2.2)	2.1 (0.7, 3.0)	0.38	0.33	0.18
	Whole	16.9 (15.7, 17.9)	16.3 (15.9, 17.3)	19.2 (17.2, 20.4)	19.2 (18.2, 19.6)	2.2 (0.8, 3.1)	2.6 (0.9, 3.6)	0.43	0.78	0.33
L4/5 (mm)	Young	17.3 (16.7, 17.7)	16.0 (15.6, 16.4)	20.2 (17.8, 20.8)	19.2 (18.6, 20.1)	3.2 (2.9, 3.5)	3.1 (2.5, 3.7)	0.02*	0.31	0.93
	Elderly	14.5 (13.2, 16.7)	14.9 (14.7, 16.4)	16.2 (14.0, 17.1)	18.5 (16.1, 18.9)	0.4 (− 0.1, 1.3)	1.8 (1.1, 2.3)	0.16	0.05	0.04*
	Whole	16.7 (14.0, 17.4)	15.9 (14.8, 16.4)	17.7 (16.4, 20.2)	18.7 (17.0, 19.5)	2.2 (0.4, 3.2)	2.3 (1.6, 3.2)	0.42	0.6	0.28
L5/S (mm)	Young	15.8 (15.0, 17.5)	15.7 (15.2, 16.1)	17.4 (16.1, 19.1)	17.3 (16.1, 18.2	1.4 (0.8, 2.3)	1.1 (0.6, 2.0)	0.35	0.29	0.34
	Elderly	13.1 (12.4, 14.2)	13.8 (13.4, 14.8)	13.6 (13.2, 14.6)	15.2 (14.6, 16.5)	0.6 (− 0.1, 1.4)	1.2 (1.0, 1.6)	0.08	0.02*	0.08
	Whole	14.6 (13.1, 15.8)	15.0 (13.8, 15.8)	15.5 (13.7, 17.4)	16.1 (15.1, 17.3)	1.0 (0.6, 1.7)	1.2 (0.7, 1.9)	0.65	0.5	0.48

Median. 1st–3rd quartile in parentheses. *Indicates statistically significant. *P*1 indicates the comparisons of the values between males and females during standing. *P*2 indicates the comparisons of the values between males and females during sitting. *P*3 indicates the comparisons between the delta value of males and females.

In summary, there was no significant difference in the change of FH between standing and sitting postures for both genders and all age groups in lumbar disc levels, except for the L4/5 level where a significant increase was observed in elderly females.

### Comparisons of the change of BBA by posture between male and female subjects (Figs. 4 and 5)

**Figure 4:**
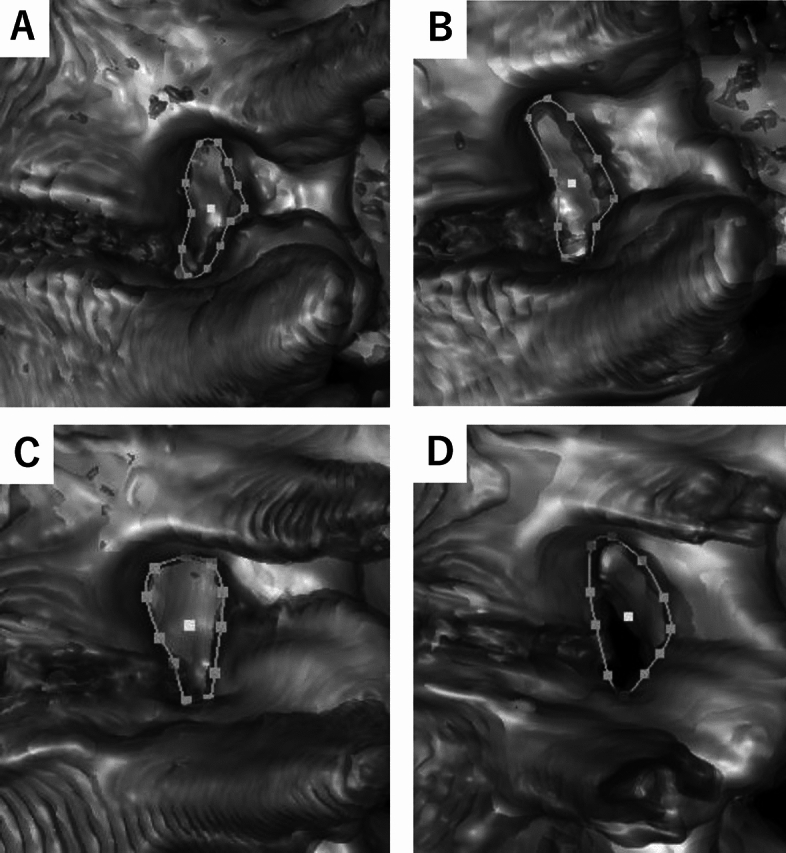
Representative Cases of the Change of Foramen by Posture in the Young Subjects. (**A**) The L4/5 foramen of 32-year-old male during standing. (**B**) The L4/5 foramen of 32-year-old male during sitting. (**C**) The L4/5 foramen of 38-year-old female during standing, (**D**) The L4/5 foramen of 38-year-old female during sitting. A significant change of BBA was observed in a female patient when transitioning to a sitting position.

**Figure 5:**
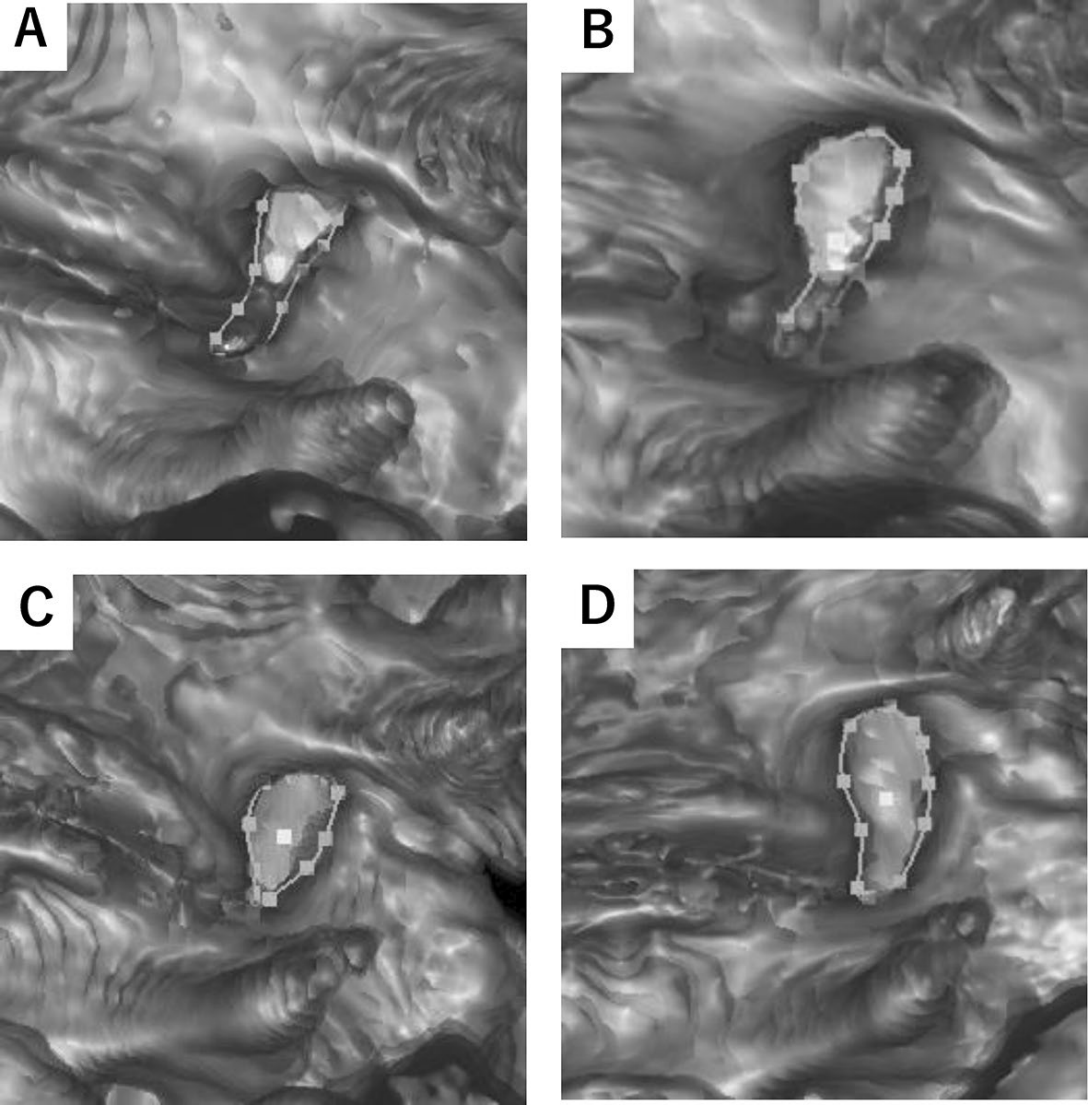
Representative cases of the change of foramen by posture in the elderly subjects. (**A**) The L4/5 foramen of 74-year-old male during standing. (**B**) The L4/5 foramen of 74-year-old male during sitting. (**C**) The L4/5 foramen of 72-year-old female during standing. (**D**) The L4/5 foramen of 72-year-old female during sitting. A significant change of FH was observed in a female patient when transitioning to a sitting position.

Similar to the findings in FH, at the upper disc levels, there were no significant differences in BBA between males and females during standing and sitting (Table 6). However, at the lower disc levels (L3/4, L4/5), females exhibited significantly larger BBA compared to males in both standing and sitting positions. Furthermore, the delta values were consistently larger in young females, with statistical significance observed in L4/5 (*p* = 0.01) and a trend observed in L3/4 in whole females (*p* = 0.08). Although the difference in the delta value between elderly males and females did not reach statistical significance, there was a trend towards a larger change in all lumbar foramen of BBA for females. These results indicate a consistent trend of larger BBA in females at the lower lumbar segments and that female tend to retain lumbar foraminal changes even in elderly age.

**Table 6:** Comparisons of the change of bone boundary area by posture between male and female subjects.

Variables		Standing		Sitting		Delta value		*P*-value		
		Male	Female	Male	Female	Male	Female	*p*1	*p*2	*p*3
L1/2 (mm^2^)	Young	94.9 (77.7, 101.1)	96.9 (85.3, 101.9)	85.0 (77.1, 104.5)	83.9 (77.7, 96.9)	4.5 (0.5, 6.6)	− 6.2 (− 17.5, 6.9)	0.32	0.82	0.12
	Elderly	64.3 (61.0, 93.3)	75.6 (64.5, 86.3)	75.9 (52.1, 105.8)	93.6 (83.9, 104.0)	− 1.3 (− 4.3, 5.9)	14.7 (2.1, 23.7)	0.15	0.09	0.05
	Whole	79.8 (58.4, 99.6)	85.9 (71.5 100.4)	81.9 (66.4, 104.7)	90.4 (80.0, 101.6)	2.2 (− 2.7, 6.6)	6.2 (− 6.7, 18.0)	0.21	0.2	0.7
L2/3 (mm^2^)	Young	87.4 (71.5, 90.3)	95.0 (74.7, 107.0)	99.7 (89.3, 109.0)	114.3 (93.5, 119.7)	10.1 (0.9, 19.9)	18.6 (1.1, 20.4)	0.23	0.17	0.82
	Elderly	75.5 (61.0, 93.3)	83.8 (72.8, 93.7)	77.3 (60.3, 116.4)	91.9 (86.0, 122.3)	1.1 (− 4.8, 3.9)	11.1 (2.2, 24.0)	0.24	0.09	0.08
	Whole	82.5 (65.9, 90.3)	88.5 (74.0, 101.5)	91.1 (70.4, 115.4)	99.4 (91.0, 120.4)	2.8 (− 2.6, 16.5)	14.2 (0.9, 22.0)	0.19	0.09	0.19
L3/4 (mm^2^)	Young	78.2 (74.7, 95.6)	87.8 (76.1, 99.5)	92.5 (89.9, 109.3)	115.3 (104.3, 132.8)	16.6 (13.9, 27.5)	30.3 (15.5, 39.1)	0.29	0.05	0.19
	Elderly	57.4 (45.9, 86.8)	85.1 (73.8, 88.7)	62.4 (49.0, 101.4)	103.0 (86.5, 118.5)	0.8 (− 1.9, 21.6)	13.2 (6.4, 31.0)	0.03*	0.01*	0.1
	Whole	76.9 (57.7, 90.1)	86.0 (74.4, 91.7)	90.4 (70.0, 109.3)	107.1 (95.2, 125.0)	15.2 (− 0.5, 24.8)	24.0 (9.2, 34.8)	0.04*	0.01*	0.08
L4/5 (mm^2^)	Young	81.1 (67.0, 95.0)	84.9 (82.0, 91.1)	95.1 (86.5, 111.8)	121.2 (101.8, 129.9)	19.4 (10.7, 31.5)	35.6 (29.2, 38.5)	0.25	0.08	0.01*
	Elderly	39.7 (30.3, 65.3)	64.3 (57.3, 71.4)	45.8 (32.4, 74.0)	75.4 (69.1, 110.7)	3.5 (− 2.0, 11.0)	11.1 (1.0, 14.3)	0.02*	0.01*	0.15
	Whole	67.0 (39.6, 82.6)	76.5 (63.1, 90.1)	80.1 (46.3, 97.8)	109.7 (74.4, 121.3)	10.8 (0.2, 25.4)	23.8 (9.9, 37.2)	0.06	0.02*	0.03*
L5/S (mm^2^)	Young	76.9 (61.6, 96.7)	82.0 (61.5, 100.5)	81.1 (67.4, 105.7)	96.2 (72.9, 113.7)	4.3 (0.9, 9.0)	5.9 (3.3, 10.9)	0.37	0.27	0.61
	Elderly	49.1 (34.8, 61.6)	61.0 (43.9, 72.9)	58.9 (35.6, 67.3)	65.6 (54.1, 84.5)	2.6 (− 1.0, 8.6)	9.4 (7.8, 11.0)	0.16	0.1	0.08
	Whole	61.6 (48.4, 85.4)	67.5 (52.2, 84.0)	67.4 (52.3, 87.3)	74.4 (56.8, 99.8)	4.2 (− 0.9, 9.0)	7.9 (3.4, 11.2)	0.26	0.15	0.07

Median. 1^st^–3rd quartile in parentheses. *Indicates statistically significant. *P*1 indicates the comparisons of the values between males and females during standing. *P*2 indicates the comparisons of the values between males and females during sitting. *P*3 indicates the comparisons between the delta value of males and females.

## Discussion

This study meticulously evaluated and compared lumbar alignment obtained from CT scans in standing and sitting positions among a group of 24 healthy young individuals and 25 elderly individuals, with both groups equally distributed between genders.

In the study, significant differences between male and female were found in LL, PI, and L3/4 BBA in standing positions, and in PT, PI, L3/4/5 BBA in sitting positions, and SS, PT, L4/5BBA when comparing the change from standing to sitting position. Among the elderly subjects, significant differences were observed in SS, PT, and L4/5 FH in changes in posture.

### Gender differences in lumbar alignment

A study by Zhou. reported the following values for standing and sitting positions in both genders (male vs. female; standing: LL: 51.1° vs. 52.0°, PT: 13.6° vs. 14.6°, sitting: LL: 26.8° vs. 21.3°, PT: 28.3° vs. 32.4°).^7^ Our study found smaller LL and PT values in both positions. This discrepancy could be attributed to the fact that our study reconstructed the sagittal plane from CT scans and involved a mixed cohort of young and elderly participants, whereas Zhou S's study focused on middle-aged and above. Moreover, the standing posture without any support may have caused subjects to be more bent forward in the standing CT, leading to different results. Hey et al. described a 50% decrease in LL and a 100% increase in PT when changing from a standing to a sitting position^8^. Another report by Hey et al. noted that in healthy young adults, the spinal alignment changes to a C-shape in a relaxed sitting position, with an 80% decrease in LL from the erect sitting position^4^. Our current study showed a decrease in LL of approximately 77% in male and 80% in female when moving from a standing to a relaxed sitting position.

Considering the changes in LL, SS, and PT due to postural changes, our results suggested that the decrease in LL and SS, and increase in PT due to the relaxed sitting position, result from the flexion of the hip joint and the tilt of the pelvis, reducing lumbar lordosis. The position of the hip joint and pelvis is fixed by the chair and foot angle, so the posterior bending of the lumbar spine that is compensated more than in the usual forward bending position is reflected.

### Assessment of intervertebral foramen area

The assessment of intervertebral foraminal area is a crucial aspect of understanding lumbar spinal biomechanics. This study assessed the intervertebral foramen area by measuring FH and BBA. Previous reports have indicated that the intervertebral foramen area and height both decreases with age^15^. In our study, we obtained similar results, demonstrating a decrease in FH and BBA with age (*p* < 0.01).

#### Consideration of axis and 3D morphology

The prior studies lack descriptions regarding the axis for intervertebral foramen measurements^5,16^. We addressed this limitation by aligning the axis towards the lower vertebral arch root and adjusting the measured area according to 3D constructed bone. This approach improved measurement accuracy and minimized inter-observer error.

#### Comparison of male and female

Previous literature lacks studies comparing the intervertebral foramen between males and females. In our study, we observed significant gender differences in sitting posture and delta BBA at L4/5, suggesting gender-specific compensatory changes in this intervertebral space. Notably, with aging, significant gender differences emerged in the angle of lordosis in the lower lumbar spine (L4 to S1), as well as delta FH. These findings imply a potential for instability in the vertical direction of vertebrae.

#### Gender-specific changes and degenerative lumbar spondylolisthesis

Our research indicated that differences in SS and PT between females and males contribute to increased stress on the lower part of the lumbar spine, notably at the L4/L5 level. This stress is pronounced due to the anatomical and biomechanical changes that occur at this segment. With the process of aging, the intervertebral foramen may diminish in size but also exhibit a shift toward the upper part of the spinal column^17^. These changes correlate with a greater incidence of degenerative lumbar spondylolisthesis in females, with the L4/L5 segment being particularly susceptible. The study further suggests that with age, alterations of trunk muscle strength and postural instability may exacerbate this condition, leading to a forward slippage of the vertebrae, especially at the cranial aspect of the spinal segment.

In conclusion, our study highlights gender differences and age-related changes in the intervertebral foramen area. The findings provide insights into gender-specific compensatory changes and their potential association with degenerative lumbar spondylolisthesis, particularly in female.

### Limitations

In this cross-sectional study, we sought to evaluate the lumbar spine components in standing and relaxed sitting positions using upright CT, with a sample comprising 24 young (aged 21–40, mean age 30.3 ± 5.7 years, 13 males, 11 females) and 25 elderly individuals (aged 61–79, mean age 65.7 ± 3.8 years, 12 males, 13 females). The selection criteria ensured a healthy cohort by excluding individuals with back pain, smokers, those outside a normal BMI range, individuals with a history of spinal surgery, non-Japanese, and those pregnant or possibly pregnant.

Despite rigorous methods, our study encounters several limitations. Firstly, the upright CT scanner's constraints on trunk motion may have affected the results, as it might not capture the full range of spinal movements. Secondly, while consistent seated postures without back support were maintained, there remains a possibility of individual variations in the "relaxed" position. Thirdly, the use of agency-recruited volunteers could introduce a selection bias, potentially affecting the representativeness of the sample. Lastly, the absence of middle-aged participants creates a gap in the data, possibly limiting the wider applicability of our findings to all age groups. Fourthly, the number of subjects might not be sufficient to determine significant differences with high statistical power, a factor that could influence the strength of our findings. Lastly, the absence of middle-aged participants creates a gap in the data, possibly limiting the wider applicability of our findings to all age groups.”

Nevertheless, this research, as a pioneering study using standing CT to detail lumbar spine components in a healthy population across genders, provides important contributions. The observed gender-based differences could enhance our understanding of conditions like degenerative spondylolisthesis.

## Conclusion

This study offers a comprehensive analysis of the detailed lumbar components in healthy individuals using standing CT, while making a comparison between genders. The findings indicate significant gender differences in the changes of pelvic tilt and sacral slope during the transition from standing to sitting, with females exhibiting a greater range of change in these parameters. This difference is particularly amplified in a sitting posture, suggesting a potential for increased stress on the lower lumbar spine. Moreover, the study highlights the changes in the area of the vertebral foramen with aging. Notably, these changes show significant gender differences, suggesting different patterns of spinal degeneration between male and female.

One key observation is the possible impact of these changes at the L4/5 level, which may explain the higher incidence of lumbar degenerative spondylolisthesis in female. As the first study of its kind, it sheds light on the physiological differences in the lumbar spine between genders and contributes to a better understanding of spinal pathologies such as degenerative spondylolisthesis.

### Supplementary Information

Supplementary Tables.

## Data Availability

Data is provided within the manuscript or supplementary information files.
