# Supplementary material for: Gender differences in spinal mobility during postural changes: a detailed analysis using upright CT
Source: Sci Rep. 2024 Apr 21;14:9154. doi: 10.1038/s41598-024-59840-8 (PMC11033253; doi:10.1038/s41598-024-59840-8)
Supplement: Supplementary file 1 — Supplementary Tables. [file 41598_2024_59840_MOESM1_ESM.docx]

**Supplemental table 1**

***Intra- and Inter-observer Reliabilities* for Various Spinal Parameters and Measurements.**

| **Variables** | **Intra-rater reliability** | **Inter-rater reliability** |
| --- | --- | --- |
| **LL** | 0.996 [0.989-0.999] | 0.986 [0.943-0.996] |
| **SS** | 0.991 [0.97-0.997] | 0.971 [0.895-0.993] |
| **PT** | 0.982 [0.940-0.995] | 0.984 [0.940-0.996] |
| **L1** | 0.995 [0.984-0.999] | 0.959 [0.850-0.990] |
| **L2** | 0.988 [0.961-0.996] | 0.947 [0.785-0.987] |
| **L3** | 0.991 [0.97-0.997] | 0.87 [0.555-0.966] |
| **L4** | 0.983 [0.946-0.995] | 0.839 [0.573-0.958] |
| **L5** | 0.974 [0.917-0.992] | 0.921 [0.718-0.980] |
| **FH** | 0.855 [0.587-0.955] | 0.805 [0.421-0.947] |
| **BBA** | 0.982 [0.942-0.995] | 0.938 [0.776-0.984] |
| 95%CI in square bracket. | | |

**Supplemental table 2**

**The P-value of the Lumber Spinal Alignment comparing between the reduction rate of young and elderly.**

| **Variables** | **P-value** | |
| --- | --- | --- |
|  | **Male** | **Female** |
| **LL (°)** | 0.01 | 0.29 |
| **SS (°)** | 0.001 | 0.39 |
| **PT (°)** | 0.0004 | 0.004 |
| **PI (°)** | 0.17 | 0.04 |
| ***Indicates statistically significant. Indicates the comparisons between the reduction rate of young and elderly.** | | |
